# Caulobacter crescentus Adapts to Phosphate Starvation by Synthesizing Anionic Glycoglycerolipids and a Novel Glycosphingolipid

**DOI:** 10.1128/mBio.00107-19

**Published:** 2019-04-02

**Authors:** Gabriele Stankeviciute, Ziqiang Guan, Howard Goldfine, Eric A. Klein

**Affiliations:** a Center for Computational and Integrative Biology, Rutgers University-Camden, Camden, New Jersey, USA; b Department of Biochemistry, Duke University Medical Center, Durham, North Carolina, USA; c Department of Microbiology, University of Pennsylvania, Philadelphia, Pennsylvania, USA; d Department of Biology, Rutgers University-Camden, Camden, New Jersey, USA; University of Chicago

**Keywords:** *Caulobacter*, glycolipids, glycosyltransferase, lipid synthesis, phosphate metabolism

## Abstract

Caulobacter crescentus adapts to phosphate starvation by elongating its cell body and a polar stalk structure. The stalk is an extension of the Gram-negative envelope containing inner and outer membranes as well as a peptidoglycan cell wall. Cellular elongation requires a 6- to 7-fold increase in membrane synthesis, yet phosphate limitation would preclude the incorporation of additional phospholipids. In the place of phospholipids, C. crescentus can synthesize several glycolipid species, including a novel glycosphingolipid (GSL-2). While glycosphingolipids are ubiquitous in eukaryotes, the presence of GSL-2 in C. crescentus is surprising since GSLs had previously been found only in *Sphingomonas* species, in which they play a role in outer membrane integrity. In this paper, we identify three proteins required for GSL-2 synthesis: CcbF catalyzes the first step in ceramide synthesis, while Sgt1 and Sgt2 sequentially glycosylate ceramides to produce GSL-2. Unlike in *Sphingomonas*, GSLs are nonessential in C. crescentus; however, the presence of ceramides does contribute to phage resistance and susceptibility to the cationic antimicrobial peptide polymyxin B. The identification of a novel lipid species specifically produced upon phosphate starvation suggests that bacteria may be able to synthesize a wider variety of lipids in response to stresses than previously observed. Uncovering these lipids and their functional relevance will provide greater insight into microbial physiology and environmental adaptation.

## Abstract (precis)

Bacteria adapt to environmental changes in a variety of ways, including altering their cell shape. Caulobacter crescentus adapts to phosphate starvation by elongating its cell body and a polar stalk structure containing both inner and outer membranes. While we generally think of cellular membranes being composed largely of phospholipids, cellular elongation occurs when environmental phosphate, and therefore phospholipid synthesis, is limited. In order to adapt to these environmental constraints, C. crescentus synthesizes several glycolipid species, including a novel glycosphingolipid. This finding is significant because glycosphingolipids, while ubiquitous in eukaryotes, are extremely rare in bacteria. In this paper, we identify three proteins required for GSL-2 synthesis and demonstrate that they contribute to phage resistance. These findings suggest that bacteria may synthesize a wider variety of lipids in response to stresses than previously observed.

## INTRODUCTION

Regulation of cell membrane composition is critical for an organism’s ability to adapt to environmental perturbations. In poikilothermic species, cells must alter their cell membrane in response to temperature changes in order to maintain relatively constant membrane fluidity. For bacteria such as Escherichia coli, cells incorporate an increasing proportion of unsaturated fatty acids as temperatures decrease (1, 2); the kinks introduced by acyl chain unsaturation decrease membrane viscosity to counteract the effects of lower temperature. Similarly, a variety of Gram-positive and Gram-negative bacteria alter the ratio of phospholipid headgroups in response to osmotic shock (3); E. coli increases the ratio of cardiolipin:phosphatidylethanolamine when osmotically stressed (4).

**FIG 1:**
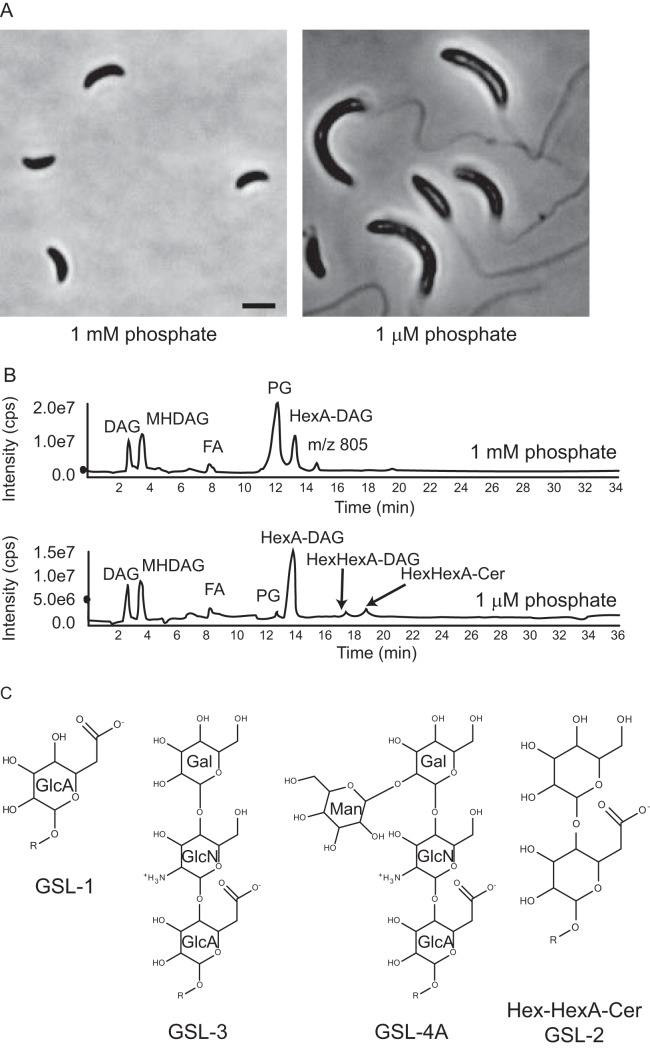
Phosphate starvation alters C. crescentus lipid composition. (A) Phase-contrast images of C. crescentus grown in HIGG with either 1 mM or 1 µM phosphate. Scale bar: 2 µm. (B) Total ion chromatograms of lipids from C. crescentus grown in HIGG with either 1 mM or 1 µM phosphate. Phosphate starvation induces the production of a HexHexA-ceramide lipid. FA, fatty acid. (C) Structures of bacterial glycosphingolipids. GSL-1, -3, and -4 are found in *Sphingomonas* species (14, 15). GSL-2 is a novel C. crescentus glycosphingolipid. The structural drawing of GSL-2 is for illustration purposes; the orientations of the hydroxyl groups have not been determined. GlcA, glucuronic acid; Gal, galactose; GlcN, glucosamine; Man, mannose; R, ceramide lipid.

Oligotrophic bacteria require adaptations to stresses associated with nutrient availability. For example, nutrient levels in freshwater lakes experience seasonal fluctuations, and phosphate concentration has been shown to be a limiting factor for bacterial growth (5). The oligotrophic Gram-negative bacterium Caulobacter crescentus responds to phosphate limitation by dramatically elongating its cell body and a polar stalk structure, a thin extension of the cell envelope, consisting of an inner membrane, a peptidoglycan cell wall, an outer membrane, and a surface layer (6) (Fig. 1A). The stalk has been hypothesized to serve as means to increase phosphate uptake (7), since all four members of the PstSCAB high-affinity phosphate import pathway are found in the stalk (7, 8). Additionally, analytical modeling of nutrient diffusion suggests that stalk elongation is the most efficient method of increasing nutrient flux to the cell while minimizing cell surface area and volume (8). Under phosphate-rich growth conditions, cells are approximately 1 µm in length, stalks are very short (∼100 nm), and phosphatidylglycerol (PG) accounts for approximately 30% of total lipids (9). Upon phosphate starvation, cell bodies and stalks can grow up to 3.5 µm and 15 µm in length, respectively, which requires significant production of new lipids to build the inner and outer membranes. When phosphate is limited it is unlikely that this new membrane contains phospholipids; therefore, we hypothesized that C. crescentus synthesizes alternative lipids for cellular and stalk elongation.

Several alphaproteobacteria adapt to phosphate limitation by increasing the production of glyceroglycolipids and ornithine lipids. For example, Agrobacterium tumefaciens synthesizes monoglucosyl diacylglycerol (DAG), glucuronosyl diacylglycerol, and diacylglycerol trimethylhomoserine (DGTS) (10, 11), while Mesorhizobium loti produces di- and triglycosyldiacylglycerols, DGTS, and ornithine lipid (12). Glycolipids make up a large proportion of the C. crescentus membrane even in phosphate-rich growth media (45% to 62%) (9), but phosphate-mediated changes in lipid composition have not been characterized. We hypothesized that during phosphate limitation C. crescentus either (i) increases the proportion of existing glycolipids or (ii) synthesizes novel lipid species to replace phospholipids.

Analysis of total membrane composition following phosphate limitation revealed that both hypotheses were correct. C. crescentus increases the amount of monohexuronosyl DAG (MHDAG) and synthesizes a novel hexosyl-hexuronosyl-ceramide glycosphingolipid (HexHexA-Cer). This glycosphingolipid (GSL) represents a novel bacterial lipid species. In this report we characterize this GSL, identify the enzymes responsible for initiating ceramide synthesis and its sequential glycosylation, and address the physiological importance of ceramide-based lipids.

## RESULTS

### Phosphate limitation induces changes in membrane composition.

Phosphate starvation induces elongation of both the cell body and stalk in C. crescentus (Fig. 1A). If we approximate the shapes of the cell body and stalk as cylinders, we can estimate that the total surface area of the cell, and thus the synthesis of membrane lipids, increases 6- to 7-fold upon phosphate limitation. This significant increase in membrane area suggests that C. crescentus must be able to produce alternatives to phospholipids during phosphate starvation. Indeed, when we compared the total lipid composition under phosphate-rich (1 mM phosphate) and phosphate-starved (1 µM phosphate) conditions by using normal phase liquid chromatography-tandem mass spectrometry (LC-MS/MS), we saw a dramatic decrease in phosphatidylglycerol and a corresponding increase in several species of glycolipids (Fig. 1B). In particular, phosphate starvation induced the synthesis of mono- and diglycosyl diacylglycerols as well as a GSL, hexose-hexuronic acid-ceramide (HexHexA-Cer; C_16_ ceramide [d18:1/16:0]) (Fig. 1B). These lipid species were identified by exact mass measurement in conjunction with collision-induced dissociation (CID) tandem mass spectrometry. The presence of a GSL was unexpected since sphingolipids, while highly abundant in eukaryotes, are rarely found in bacteria. Some species in the *Bacteroides*, *Porphyromonas*, and *Prevotella* genera are capable of synthesizing the phosphosphingolipids ceramide phosphorylethanolamine and/or ceramide phosphoglycerol (13). Non-phosphate-containing GSLs have been described only for the family *Sphingomonadaceae*, in which they can function as a substitute for lipopolysaccharide (LPS) (14). Structural analyses of *Sphingomonas* GSLs have revealed carbohydrate moieties containing 1, 3, or 4 sugar units (Fig. 1C) (14, 15). In contrast, the GSL found in C. crescentus has two sugars and thus represents a novel bacterial GSL species, which we named GSL-2 (Fig. 1C).

### Ceramide synthesis in C. crescentus.

Sphingolipid synthesis begins with the production of a ceramide molecule which is then modified with various polar groups. In eukaryotes, ceramide synthesis is a four-step process that begins with the condensation of serine and a fatty acyl coenzyme A (acyl-CoA) to produce 3-oxo-sphinganine in a reaction catalyzed by oxoamine synthase (16). While each of the enzymatic steps in ceramide synthesis has been well characterized in eukaryotes (17), only the oxoamine synthase enzyme required for the first step appears to be conserved in bacteria. Indeed, bacterial species have been identified encoding one or more oxoamine synthases, including serine palmitoyltransferase (Spt), 8-amino-7-oxononanoate synthase (BioF), 5-aminolevulinate synthase (HemA), and 2-amino-3-oxobutyrate coenzyme A ligase (Kbl) (18). C. crescentus encodes three putative oxoamine synthases: CCNA_01220 (BioF), CCNA_01417 (HemA), and CCNA_01647 (BioF).

**FIG 2:**
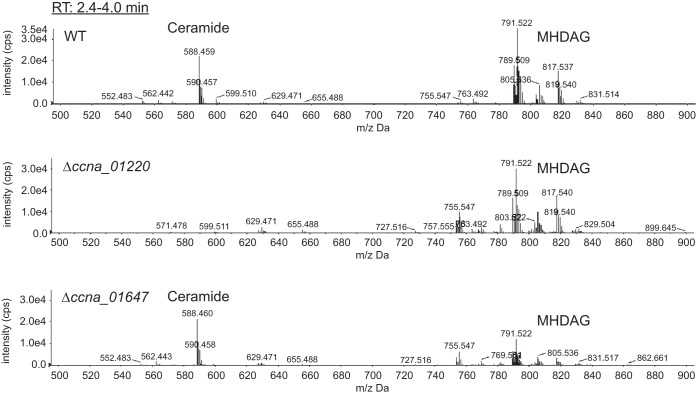
CCNA_01220 is required for ceramide synthesis. Wild-type, Δ*ccna_01220*, and Δ*ccna_01647* cells were grown in HIGG–1 µM phosphate, and total lipids were isolated. Negative-ion ESI/MS shows the [M + Cl]^−^ ions of the lipids (ceramide and MHDAG) emerging at 2.4 to 4.0 min.

To assess the role of the candidate oxoamine synthases in ceramide synthesis, total lipid composition was analyzed in wild-type, Δ*ccna_01220*, and Δ*ccna_01647* cells. We were unable to obtain a deletion of *ccna_01417* since this is an essential gene (19); however, recent biochemical analysis of CCNA_01417 reveals that it is most likely involved in the production and regulation of heme cofactors (20). Ceramides were completely absent in the Δ*ccna_01220* strain (Fig. 2); therefore, we refer to this gene as *ccbF*, for Caulobacter crescentus BioF. Complementation of the *ccbF* deletion restored ceramide synthesis (see Fig. S1A in the supplemental material). Interestingly, deletion of BioF homolog CCNA_01647 had no effect on ceramide synthesis despite the fact that the protein has 51% similarity and 35% identity to CcbF (Fig. 2). We note that while the *ccna_01647* deletion did not affect ceramide levels, we observed a reduction in MHDAG synthesis (Fig. 2), though the mechanism for this decrease is unknown.

10.1128/mBio.00107-19.6 FIG S1 LC/MS confirmation of gene complementation. Download FIG S1, PDF file, 0.3 MB. Copyright © 2019 Stankeviciute et al. 2019 Stankeviciute et al. This content is distributed under the terms of the Creative Commons Attribution 4.0 International license.

### Glycosphingolipid synthesis requires two sequential glycosyltransferases.

**FIG 3:**
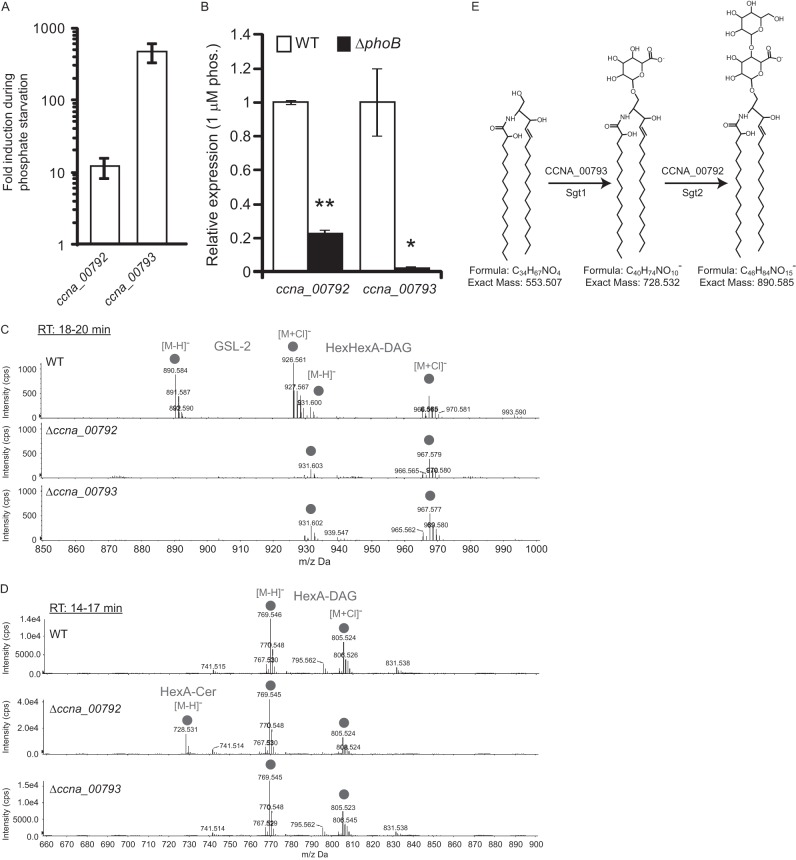
Identification of sphingolipid glycosyltransferases. (A) qRT-PCR of candidate glycosyltransferase genes *ccna_00792* and *ccna_00793* was performed on samples from C. crescentus grown in HIGG with 1 mM or 1 µM phosphate. The graph represents the fold induction seen in 1 µM phosphate relative to 1 mM phosphate. mRNA levels are normalized to *rpoD* expression. Error bars indicate the SEs (*n* = 3). (B) RNA was extracted from wild-type and Δ*phoB* cells grown in HIGG–1 μM phosphate and analyzed by qRT-PCR. mRNA levels are normalized to *rpoD* expression. Error bars indicate the SEs (*n* = 3). *, *P* = 0.04; **, *P* < 10^−5^ (two-tailed *t* test). (C and D) Wild-type, Δ*ccna_00792*, and Δ*ccna_00793* cells were grown in HIGG–1 µM phosphate, and total lipids were isolated. Negative-ion ESI/MS shows the [M − H]^−^ and [M + Cl]^−^ ions of the lipids emerging at 18 to 20 min (C) and 14 to 17 min (D). (E) The LC/MS data support a model of successive glycosylation of ceramide to HexA-Cer and HexHexA-Cer by Sgt1 and Sgt2, respectively. The structural drawings are for illustration purposes; the orientations of the hydroxyl groups have not been determined.

GSL-2 has a novel glycosylation pattern consisting of a hexose and a hexuronic acid (Fig. 1C). Lipid glycosylation, in both eukaryotes and prokaryotes, is performed by GT-4 family glycosyltransferases; C. crescentus encodes 11 putative GT-4 glycosyltransferases (21). To identify the glycosyltransferases required for GSL-2 synthesis, we narrowed down the list of candidate genes using the following criteria: (i) genes upregulated upon phosphate starvation (22), (ii) nonessential genes (19), and (iii) genes without a direct homolog in E. coli, since E. coli does not produce GSLs. Of the 11 initial candidates, only 2 genes fit all three criteria: *ccna_00792* and *ccna_00793* (Fig. 3A and Table S4). Both candidate genes are part of the PhoB regulon, which contains genes upregulated under phosphate starvation conditions (Fig. 3B) (22). Deletion of *ccna_00792* or *ccna_00793* resulted in a complete lack of diglycosylated ceramide (Fig. 3C), whereas deleting *ccna_00792* led to the accumulation of the monoglycosylated HexA-Cer species (Fig. 3D). Complementation of these deletions recovered ceramide glycosylation (Fig. S1B and C). These data support a model of sequential ceramide glycosylation by CCNA_00793 and CCNA_00792, which we are naming sphingolipid glycosyltransferases 1 and 2 (Sgt1 and -2), respectively (Fig. 3E). Sgt1 functions as a glucuronosyltransferase, adding a hexuronic acid, while Sgt2 is a glycosyltransferase responsible for adding a hexose sugar. Sgt1 and -2 appear to specifically glycosylate sphingolipids, as neither deletion affected the glycosylation of DAG-based lipids (Fig. 3C and D). The specificity of Sgt1 and -2 was further confirmed by heterologous expression in E. coli, in which we could detect only nonglycosylated lipids (Fig. S2A and B).

10.1128/mBio.00107-19.5 TABLE S4 Selection criteria for assessing candidate glycosyltransferases. Download Table S4, DOCX file, 0.01 MB. Copyright © 2019 Stankeviciute et al. 2019 Stankeviciute et al. This content is distributed under the terms of the Creative Commons Attribution 4.0 International license.

10.1128/mBio.00107-19.7 FIG S2 Expression of Sgt1 and -2 in E. coli. Download FIG S2, PDF file, 0.8 MB. Copyright © 2019 Stankeviciute et al. 2019 Stankeviciute et al. This content is distributed under the terms of the Creative Commons Attribution 4.0 International license.

**FIG 4:**
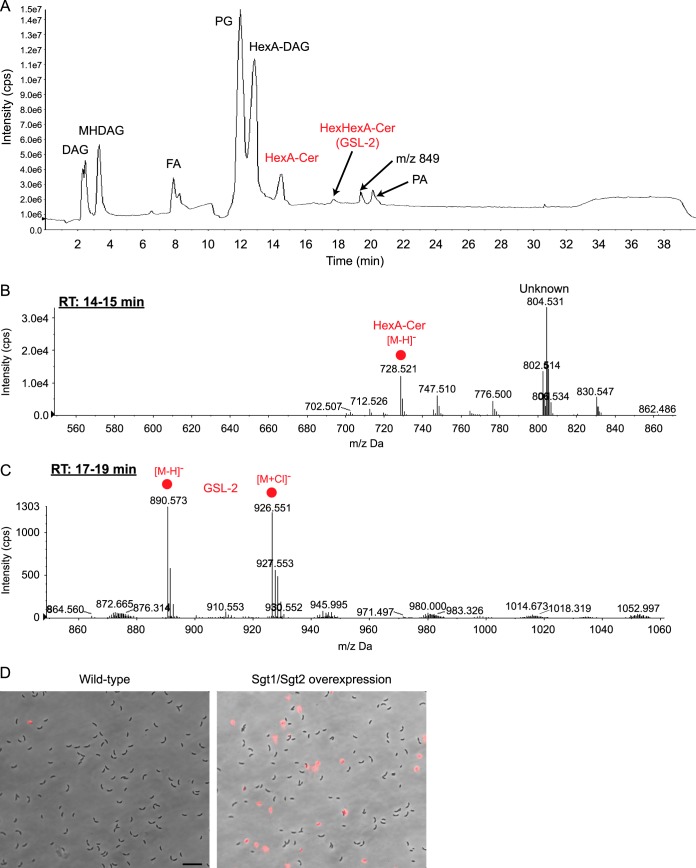
Ectoptic production of glycosphingolipids is detrimental to cell integrity. (A to C) Lipids were analyzed from strain GS81 (Sgt1 and Sgt2 overexpression) grown in HIGG–1 mM phosphate and induced with 0.3% xylose and 0.5 mM vanillate. (A) The total ion chromatogram shows the simultaneous production of phospholipids and glycosphingolipids. Negative-ion ESI/MS showing the [M − H]^−^ and [M + Cl]^−^ ions of the lipids emerging at 14 to 15 min (B) and 17 to 19 min (C) confirms the synthesis of HexA-Cer and GSL-2. We note the presence of an unknown lipid species with *m/z *=* *804.531. (D) Wild-type and Sgt1 and -2 overexpression cells were grown in HIGG–1 mM phosphate and induced with 0.3% xylose and 0.5 mM vanillate. Cells were labeled with 1 µg/ml of propidium iodide and imaged by fluorescence microscopy. The micrographs are overlays of phase and fluorescence images. Scale bar: 10 µm.

While GSL-2 synthesis occurs in response to phosphate starvation, neither ceramides nor GSL-2 appears to be necessary for stalk biogenesis or cell elongation (Fig. S3A). This is likely due to the sufficiency of upregulated DAG lipids under low-phosphate conditions (Fig. 1B). Unlike GSL-2, nonglycosylated ceramides are produced across a wide range of phosphate concentrations, albeit at lower levels in the presence of excess phosphate (Fig. S3B). Surprisingly, *ccbF* mRNA levels are reduced during phosphate starvation, despite higher levels of ceramide production (Fig. S3C). Restriction of ceramide glycosylation to growth environments in which phospholipid synthesis is limited appears to be critical for membrane homeostasis. Overexpression of Sgt1 and Sgt2 in high-phosphate media results in the production of both HexA-Cer and GSL-2 (Fig. 4A to C). Unlike physiological glycosphingolipid production during phosphate starvation, Sgt1 and -2 overexpression leads to an accumulation of HexA-Cer (compare Fig. 1B to Fig. 4A). Furthermore, we did not detect HexHexA-DAG under these conditions, providing additional evidence that Sgt1 and -2 specifically glycosylate ceramide lipids (Fig. 4C). The production of glycosphingolipids under high-phosphate conditions leads to cell lysis, as assessed by propidium iodide staining (Fig. 4D). This phenomenon may be due to an excess of anionic lipids and is consistent with our previous observation that overabundance of anionic phospholipids is detrimental in E. coli (23).

10.1128/mBio.00107-19.8 FIG S3 Ceramides are not required for stalk or cell body elongation. Download FIG S3, EPS file, 1.6 MB. Copyright © 2019 Stankeviciute et al. 2019 Stankeviciute et al. This content is distributed under the terms of the Creative Commons Attribution 4.0 International license.

### Ceramides regulate antibiotic and phage susceptibility.

**FIG 5:**
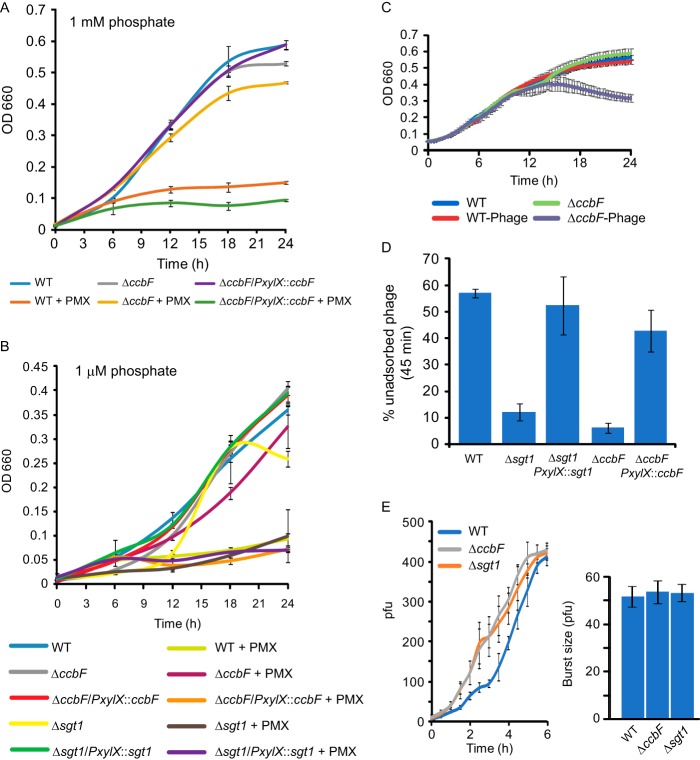
Ceramides regulate polymyxin B resistance and phage susceptibility. (A and B) Growth curves for the indicated strains were measured in HIGG with 1 mM phosphate (A) or 1 µM phosphate (B). Cells were grown in the presence of polymyxin B (PMX; 10 µg/ml) or water (control). Complementation strains were induced with 0.3% xylose. Error bars indicate the SEs (*n* = 3). (C) Growth curves for wild-type and Δ*ccbF* cells were measured in PYE in the presence of phage ΦCr30 (5 × 10^5^ PFU/ml) or water (control). Error bars indicate the SEs (*n* = 3). (D) Phage adsorption was measured for the indicated strains in HIGG–1 µM phosphate. Complementation strains were induced with 0.3% xylose. Error bars indicate the SEs (*n* ≥ 4). (E) Phage burst size was measured for the indicated strains in HIGG–1 µM phosphate. Phage titer was recorded over time (left), and the fold increase from the initial titer was calculated as the burst size (right). Error bars indicate the SEs (*n* = 3).

Under laboratory growth conditions, ceramides and GSLs are nonessential; cell growth is minimally perturbed in *ccbF* and *sgt1* deletion strains whether grown in high-phosphate (1 mM) or low-phosphate (1 µM) minimal medium (Fig. 5A and B). GSL-producing *Sphingomonas* species are resistant to the antibiotic polymyxin B (24); therefore, we tested whether ceramides or GSL-2 conferred similar resistance on C. crescentus. Surprisingly, the deletion of *ccbF* increased resistance to polymyxin B under both high- and low-phosphate conditions (Fig. 5A and B). Under low-phosphate conditions, the Δ*sgt1* strain did not have increased polymyxin B resistance, demonstrating that the resistance phenotype was specifically due to the presence of ceramide lipids regardless of their glycosylation (Fig. 5B). Under both phosphate conditions, complementation of *ccbF* restored sensitivity to polymyxin B (Fig. 5A and B).

Modifying the bacterial envelope structure and composition can also affect cellular interactions with bacteriophages. Phage ΦCr30 infects C. crescentus by attaching to the extracellular surface layer (S-layer) (25). Growth curves of wild-type and *ccbF* deletion strains infected with ΦCr30 in peptone-yeast extract (PYE) demonstrated that ceramides are important for increasing phage resistance (Fig. 5C); this initial screen was performed in PYE because ΦCr30 infections are generally inhibited in minimal media (26). The increased susceptibility of the Δ*ccbF* strain could be attributed to either enhanced phage adsorption or an increased viral burst size. We tested the ability of ΦCr30 to adsorb to wild-type, Δ*ccbF*, and Δ*sgt1* cells in Hutner base-imidazole-glucose-glutamate medium (HIGG)–1 µM phosphate. Both of the mutant strains had an enhanced rate of phage adsorption which was restored to normal upon complementation (Fig. 5D), suggesting that mature GSL-2 is required to inhibit phage adsorption. Measurements of burst size did not reveal any differences between the strains in low-phosphate medium (Fig. 5E); however, the *ccbF* and *sgt1* deletion strains appear to have shorter latent periods, consistent with faster phage adsorption to these cells (Fig. 5E).

Phage ΦCr30 attaches to C. crescentus by binding to the cell envelope S-layer, a crystalline lattice composed of the protein RsaA (27). The S-layer is, in turn, anchored to the cell through interactions with the O-antigen domain of lipopolysaccharide (LPS) (28). A comparison of LPS and S-layer production in wild type and GSL mutants did not reveal any remarkable distinctions between the strains (Fig. S4A and B), suggesting that the enhanced phage adsorption observed in the GSL mutants is not due to an increase in S-layer production. The accessibility of the S-layer to phage is restricted by the production of an exopolysaccharide (EPS) capsule (29). To test whether the absence of ceramides disrupts EPS production, C. crescentus strains were grown in HIGG–1 µM phosphate and incubated with fluorescein isothiocyanate (FITC)-labeled dextran (29). The wide zone of exclusion around the wild-type and Δ*ccbF* cells show that they produce EPS, in contrast to the non-EPS-producing ΔMGE strain (29) (Fig. S4C).

10.1128/mBio.00107-19.9 FIG S4 Depletion of GSLs does not affect LPS, S-layer, or EPS production. Download FIG S4, PDF file, 0.2 MB. Copyright © 2019 Stankeviciute et al. 2019 Stankeviciute et al. This content is distributed under the terms of the Creative Commons Attribution 4.0 International license.

## DISCUSSION

C. crescentus adapts to phosphate limitation, in part, by dramatically elongating both its cell body and polar stalk appendage (7, 30) (Fig. 1A), requiring a significant amount of lipid synthesis. Without the environmental phosphate required for phospholipid synthesis, C. crescentus upregulates the production of several glycolipid species, including a novel glycosphingolipid, GSL-2 (Fig. 1B). In this study, we identified three enzymes involved in GSL production: CcbF is responsible for the first step of ceramide synthesis (Fig. 2), while Sgt1 and Sgt2 sequentially glycosylate ceramide to yield GSL-2 (Fig. 3C to E).

Upregulation of glycolipid synthesis in response to phosphate limitation has been previously described for Agrobacterium tumefaciens and Mesorhizobium loti (11, 12). In these species, cells produce nonphosphorus glycosyl-DAGs. While C. crescentus also produces mono- and diglycosyl-DAGs, this is the first demonstration of bacterial GSL synthesis in response to phosphate starvation. While GSLs are found ubiquitously in eukaryotic organisms, their presence in bacteria was thought to be limited to species of the family *Sphingomonadaceae*. In *Sphingomonas* species, GSLs are used as a substitute for LPS in the outer membrane and contain 1, 3, or 4 sugar units (14, 15). Sphingomonas wittichii strain RW1 produces two different monoglycosylated GSLs in place of LPS (31). Not surprisingly, the gene for serine palmitoyltransferase, which catalyzes the first step of ceramide synthesis, is an essential gene in S. wittichii (32). In contrast, C. crescentus GSL synthesis genes are nonessential and GSL-2 is produced even in the presence of LPS (Fig. S4A). Furthermore, ablation of ceramide or GSL-2 has no effect on proliferation (Fig. 5A and B) or cellular elongation (Fig. S3A). Thus, we conclude that while GSL production occurs under conditions of phosphate limitation, it is dispensable for cell elongation, stalk synthesis, and survival. This is likely due to the presence of sufficient glycosylated DAGs to compensate for the loss of GSLs.

This study is the first to identify bacterial glycosyltransferase enzymes required for ceramide glycosylation. As expected, BLAST homology searches (33) demonstrate that outside the *Caulobacteraceae* family, Sgt1 and Sgt2 are most homologous to glycosyltransferases in the GSL-producing *Sphingomonadaceae* family. Unlike many other bacterial glycosyltransferases which demonstrate a high degree of promiscuity regarding sugar acceptors (34, 35), Sgt1 appears to have a high degree of specificity toward ceramide glycosylation. Deletion of *sgt1* in C. crescentus has no effect on glycosyldiacylglycerol production (Fig. 3C), and heterologous expression of Sgt1 and Sgt2 in E. coli does not lead to lipid glycosylation (Fig. S2).

While *Sphingomonas* species use GSLs to replace LPS, the role of GSLs in C. crescentus is less clear. Ceramide synthesis occurs over a wide range of phosphate concentrations, yet mature GSLs are produced only during phosphate starvation. Complete deletion of ceramides appears to alter the function of C. crescentus membranes, resulting in increased resistance to the lipid-interacting antibiotic polymyxin B and increased sensitivity to phage-mediated killing (Fig. 5A to C). These effects occur despite the absence of gross changes to LPS, S-layer, or EPS production (Fig. S4A to C). Resistance to cationic antimicrobial peptides like polymyxin B often occurs by reducing the negative charge of the membrane to prevent binding; for example, in E. coli, lipid A is modified with 4-amino-4-deoxy-l-arabinose (36) to neutralize charge. In C. crescentus, the impact of ceramide or GSL deficiency on total membrane charge is less clear; nonglycosylated ceramides are neutral, while the hexuronic acid found in GSL-2 is anionic. Therefore, the relative abundances of all lipid species would be required to assess the role of membrane charge in antibiotic resistance.

The increased susceptibility of ceramide-depleted cells to phage lysis appears to be due to enhanced phage adsorption to the *ccbF* and *sgt1* deletion strains (Fig. 5D). Increased adsorption reduces the phage latency period without affecting the phage burst size (Fig. 5E). Although the abundance of S-layer protein was not affected in the GSL mutants (Fig. S4B), recent biophysical studies have shown that the S-layer protein RsaA can exist on the cell surface in either a crystalline or aggregated state (37). This is consistent with cryo-electron tomography showing distinct regions of S-layer organization in intact C. crescentus cells (38). While we do not know exactly how phage ΦCr30 binds to the S-layer, it is possible that GSLs affect S-layer organization, rather than production, thereby regulating phage interactions.

## MATERIALS AND METHODS

### Bacterial strains, plasmids, and growth conditions.

The strains, plasmids, and primers used in this study are described in Tables S1, S2, and S3, respectively. Details regarding strain construction are available in the supplemental materials. C. crescentus wild-type strain NA1000 and its derivatives were grown at 30°C in peptone-yeast extract (PYE) medium (39) for routine culturing. To control phosphate levels, C. crescentus was grown in Hutner base-imidazole-glucose-glutamate media (HIGG) with variable amounts of phosphate (1 to 1,000 µM) (40). E. coli strains were grown at 37°C in LB medium. When necessary, antibiotics were added at the following concentrations: kanamycin, 30 µg/ml in broth and 50 µg/ml in agar (abbreviated 30:50) for E. coli and 5:25 for C. crescentus; ampicillin, 50:100 for E. coli; tetracycline, 12:12 for E. coli and 1:2 for C. crescentus; gentamicin, 15:20 for E. coli and 0.5:5 for C. crescentus; and spectinomycin, 50:50 for E. coli and 25:100 for C. crescentus. Gene expression was induced in C. crescentus with either 0.3% (wt/vol) xylose or 0.5 mM vanillate. E. coli gene expression was induced with isopropyl-β-d-1-thiogalactopyranoside (IPTG; 1 mM). Phage titering was performed by adding 1 to 10 µl of ΦCr30 to 100 µl of an overnight culture of NA1000 in PYE. This mixture was added to 4 ml of soft agar (0.3% [wt/vol] agar in PYE) and overlaid on a PYE-agar plate. After solidifying, the plate was incubated overnight at 30°C and plaques were counted.

10.1128/mBio.00107-19.2 TABLE S1 List of strains used in this study. Download Table S1, DOCX file, 0.02 MB. Copyright © 2019 Stankeviciute et al. 2019 Stankeviciute et al. This content is distributed under the terms of the Creative Commons Attribution 4.0 International license.

10.1128/mBio.00107-19.3 TABLE S2 List of plasmids used in this study. Download Table S2, DOCX file, 0.02 MB. Copyright © 2019 Stankeviciute et al. 2019 Stankeviciute et al. This content is distributed under the terms of the Creative Commons Attribution 4.0 International license.

10.1128/mBio.00107-19.4 TABLE S3 List of primers used in this study. Download Table S3, DOCX file, 0.02 MB. Copyright © 2019 Stankeviciute et al. 2019 Stankeviciute et al. This content is distributed under the terms of the Creative Commons Attribution 4.0 International license.

### Microscopy and image analysis.

Cells were spotted onto 1% agarose pads made in the corresponding growth medium. Phase microscopy was performed on a Nikon TiE inverted microscope equipped with a Prior Lumen 220PRO illumination system, Zyla sCMOS 5.5-megapixel camera, CFI Plan Apochromat 100× oil immersion objective (numerical aperture [NA] of 1.45 and working distance [WD] of 0.13 mm), and NIS Elements software for image acquisition. Cell and stalk dimensions were measured using *Morphometrics* (41) and ImageJ v. 1.48q (NIH), respectively. To measure membrane permeability, cells were grown in the presence of 1 µg/ml of propidium iodide. EPS production was assessed as previously described (29). Briefly, 500 microliters of cells grown in HIGG–1 µM phosphate were collected by centrifugation (14,000 × *g*, 5 min), and the pellet was resuspended in 30 µl of 0.5× phosphate-buffered saline (PBS). Ten microliters of the cell suspension was mixed with 5 µl of FITC-dextran (10 mg/ml; molecular weight [MW], 2 MDa; Sigma) and 1 µl of SlowFade Diamond mountant (Thermo Scientific). Two microliters of this mixture was spotted onto a glass slide, coverslipped, and sealed with vaseline-lanolin-paraffin (VALAP; 1:1:1) for imaging.

### qRT-PCR.

RNA was extracted from bacterial cultures using the Qiagen RNeasy kit. Following DNase digestion, RNA (5 ng/µl) was reverse transcribed using a high-capacity cDNA reverse transcription (RT) kit (Applied Biosystems). One microliter of cDNA was used as a template in a 10-µl quantitative RT-PCR (qRT-PCR) performed with Power SYBR reagent (Applied Biosystems). qRT-PCR was performed on an ABI QuantStudio 6 using the threshold cycle (ΔΔ*C_T_*) method. *rpoD* expression was used as the loading control.

### Lipid extraction.

C. crescentus strains were grown in 500 ml of HIGG with either 1 mM or 1 µM phosphate until reaching stationary phase. Sgt1 and -2 E. coli expression strains were grown overnight in 500 ml of LB medium with 1 mM IPTG to induce protein expression. Lipids were extracted by the method of Bligh and Dyer (42). Cells were harvested in glass tubes at 10,000 × *g* for 30 min, and the majority of the supernatant was removed; stalked C. crescentus organisms are very buoyant and do not form tight pellets, preventing the complete removal of supernatant. The cells were resuspended in the residual supernatant, 3.75 volumes of 1:2 (vol/vol) chloroform-methanol was added, and the samples were mixed by vortexing. Chloroform (1.25 volumes) and water (1.25 volumes) were added sequentially with vortexing to create a two-phase system, and the samples were centrifuged at 200 × *g* for 5 min at room temperature. The bottom, organic phase was transferred to a clean tube with a Pasteur pipette and washed twice in “authentic” upper phase. Subsequently, the residual organic phase with the lipids was collected and dried under argon.

### LC-ESI-MS/MS.

Methods for liquid chromatography-electrospray ionization-tandem mass spectrometry (LC-ESI-MS/MS) have been described previously (43, 44). Briefly, normal phase LC was performed on an Agilent 1200 quaternary LC system equipped with an Ascentis Silica high-performance liquid chromatography (HPLC) column, 5 µm, 25 cm by 2.1 mm (Sigma-Aldrich, St. Louis, MO). The LC eluent (with a total flow rate of 300 µl/min) was introduced into the ESI source of a high-resolution TripleTOF5600 mass spectrometer (Applied Biosystems, Foster City, CA). Instrumental settings for negative-ion ESI and MS/MS analysis of lipid species were as follows: IS, −4,500 V; CUR, 20 lb/in^2^; GSI, 20 lb/in^2^; DP, −55 V; and FP, −150 V. The MS/MS analysis used nitrogen as the collision gas. Data analysis was performed using Analyst TF1.5 software (Applied Biosystems).

### Growth curve analysis.

For polymyxin B sensitivity assays, C. crescentus cells were diluted to an optical density at 660 nm (OD_660_) of 0.05 in HIGG with 1 mM or 1 µM phosphate, treated with 30 µg/ml of polymyxin B, and incubated with shaking (250 rpm and 30°C). Complementation gene expression was induced with 0.3% xylose. Samples were taken at the desired times for absorbance measurements (OD_660_). For phage sensitivity assays, cells were grown in PYE and diluted to an OD_660_ of 0.05. A total of 148 µl of culture was dispensed per well in a 96-well plate. To each well, 2 µl of water (control) or ΦCr30 (final concentration, 5 × 10^5^ PFU/ml) was added. To prevent evaporation, each well was overlaid with 100 µl of mineral oil. OD_660_ was recorded every 20 min on a BMG Labtech CLARIOstar plate reader with incubation at 30°C with continuous shaking.

### Phage adsorption and burst size quantification.

Phage adsorption and burst size were measured essentially as previously described (25). To measure phage adsorption, cells were grown in HIGG–1 µM phosphate and diluted to an OD_660_ of 0.2. Cells (1 ml) were aliquoted into glass culture tubes, and 10^5^ PFU of ΦCr30 was added. Cultures were incubated with shaking at 30°C; at various time points, 10 µl of culture was removed and diluted into 1 ml of water-chloroform (9:1 [vol/vol]). Ten microliters of this mixture was used to titer the unbound phage as described above. To measure viral burst size, cells were grown in HIGG–1 µM phosphate and diluted to an OD_660_ of 0.1. Cells (0.5 ml) were infected with 0.5 × 10^5^ PFU of ΦCr30 and incubated at 30°C for 15 min. The culture was diluted 1,000-fold into HIGG–1 µM phosphate; a 200-µl aliquot was removed every 15 min for titering as described above.

### SDS-PAGE and protein staining.

E. coli strains were grown overnight in LB medium with 1 mM IPTG to induce protein expression. A total of 500 µl of each strain was collected by centrifugation (6,000 × *g* for 2 min) and the pellet was resuspended in 100 µl of sample buffer. Protein samples were resolved on a 12% SDS-PAGE gel and stained with Coomassie blue for visualization.

### LPS purification and analysis.

Lipopolysaccharide (LPS) was purified essentially as previously described (45). Briefly, 5 ml of C. crescentus cells grown in HIGG–1 µM phosphate (OD_660_ = 0.5) was collected and washed once in 10 mM HEPES (pH 7.2). Cells were resuspended in 250 µl of TE buffer (10 mM Tris, 1 mM EDTA [pH 7.2]) and frozen overnight at −20°C. Cells were thawed, treated with 1 µl of DNase (0.5 mg/ml), 20 µl of lysozyme (10 mg/ml), and 3 µl of MgCl_2_ (1 M), and incubated at room temperature for 15 min. For each sample, 36.25 µl was mixed with 12.5 µl of 4× SDS sample buffer and boiled at 100°C for 10 min. After cooling to room temperature, 1.25 µl of proteinase K (20 mg/ml) was added and samples were incubated at 60°C for 1 h. LPS samples were resolved on a 12% SDS-PAGE gel and stained with a Pro-Q Emerald 300 LPS stain kit according to the manufacturer’s protocol (Thermo Scientific). Images were acquired on a Bio-Rad ChemiDoc MP using UV excitation and a 530-nm emission filter.

### S-layer (RsaA) purification and analysis.

RsaA was purified essentially as previously described (46). Briefly, cells were grown overnight in HIGG–1 µM phosphate and 5 ml (OD_660_ = 0.6) was collected by centrifugation. The cell pellets were washed twice in 5 ml of 10 mM HEPES (pH 7.2), resuspended in 200 µl of 100 mM HEPES (pH 2), and incubated at room temperature for 10 min. Cells were pelleted (10 min at 5,000 × *g*), and the supernatant containing RsaA was collected and neutralized with 2.8 µl of 10 N NaOH. RsaA samples in 1× sample buffer were resolved on a 7.5% SDS-PAGE gel without heat denaturing and stained with Krypton protein stain (Thermo Scientific). Images were acquired on a Bio-Rad ChemiDoc MP using green light-emitting diode (LED) excitation and a 605-nm emission filter.

10.1128/mBio.00107-19.1 TEXT S1 Detailed description of strain construction and supplemental references. Download Text S1, DOCX file, 0.03 MB. Copyright © 2019 Stankeviciute et al. 2019 Stankeviciute et al. This content is distributed under the terms of the Creative Commons Attribution 4.0 International license.
