# Supplementary material for: Caulobacter crescentus Adapts to Phosphate Starvation by Synthesizing Anionic Glycoglycerolipids and a Novel Glycosphingolipid
Source: mBio. 2019 Apr 2;10(2):e00107-19. doi: 10.1128/mBio.00107-19 (PMC6445935; doi:10.1128/mBio.00107-19)
Supplement: FIG S1 [file mBio.00107-19-sf001.pdf]

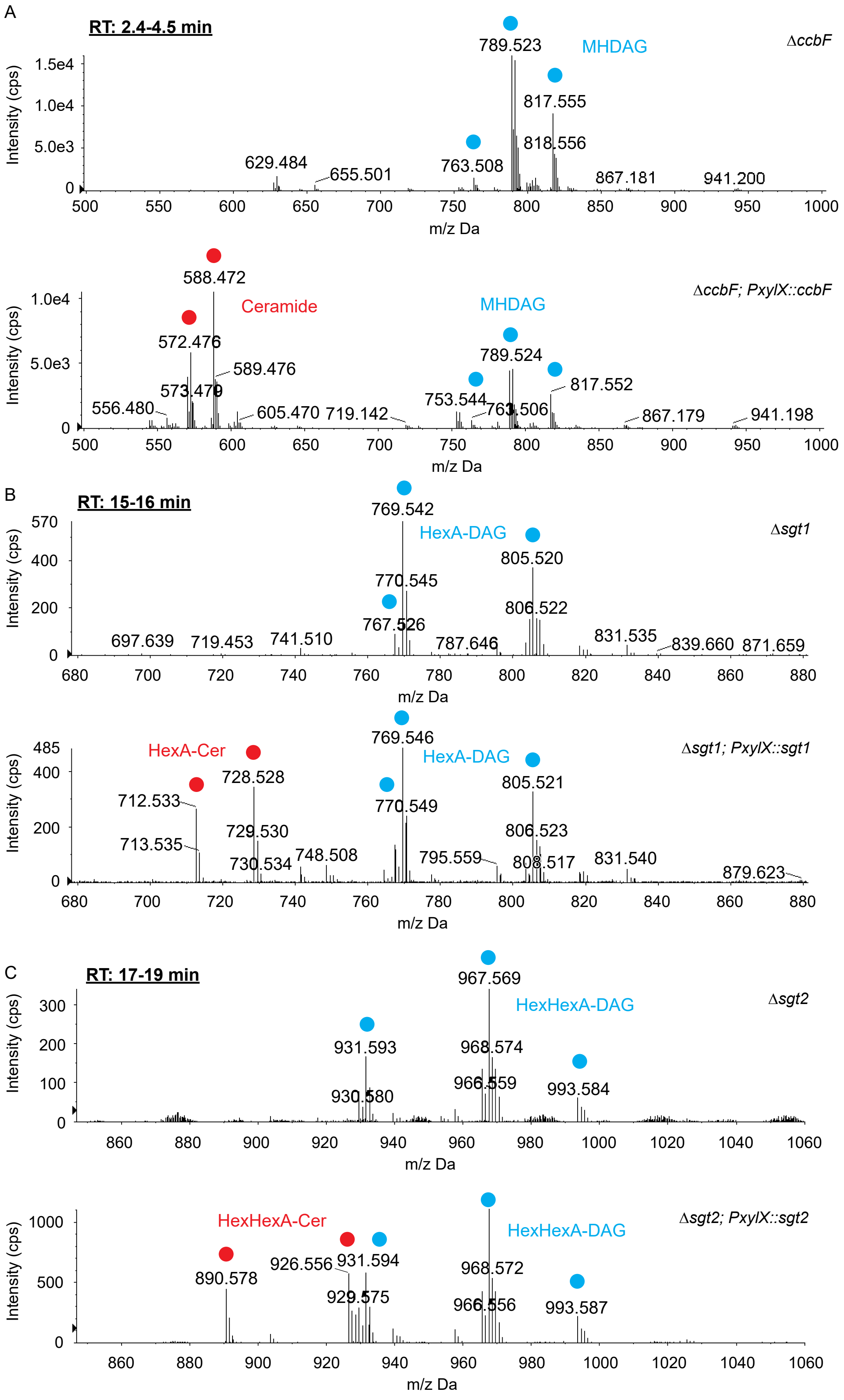

Figure S1. Genetic complementation restores ceramide and GSL synthesis. (A-C) Lipids were extracted from the indicated strains and analyzed by LC/MS. For the complementation strains, gene expression was induced with 0.3% xylose. Negative ion ESI/MS showing lipid ions confirms the synthesis of ceramide (A), HexA-Cer (B), and GSL-2 (C). We note that both the ceramide and HexA-Cer complementaion samples contain an unexplained peak 16 Da smaller than the parent ion indicating the loss of an oxygen atom.
