# Supplementary material for: Caulobacter crescentus Adapts to Phosphate Starvation by Synthesizing Anionic Glycoglycerolipids and a Novel Glycosphingolipid
Source: mBio. 2019 Apr 2;10(2):e00107-19. doi: 10.1128/mBio.00107-19 (PMC6445935; doi:10.1128/mBio.00107-19)
Supplement: TABLE S4 [file mBio.00107-19-st004.docx]

**Table S4. *C. crescentus* GT-4 family glycosyltransferases.** Regulation by phosphate starvation and essentiality are based on previously published studies (7,8). Closest *E. coli* homologues were determined by BLAST (9) against strain MG1655. An E-value cutoff of 10^-3^ and query coverage of >40% were used as the criteria for identifying homologues. To perform the reverse BLAST, the identified *E. coli* homologues were BLASTed against the *C. crescentus* NA1000 genome. The results were scored as positive if the top hit was the same protein used to find the *E. coli* homologue.

| **Gene** | **Regulated by phosphate starvation (7)** | **Essential (8)** | **Closest *E. coli* homolog** | **Reverse BLAST** |
| --- | --- | --- | --- | --- |
| *ccna_00469* | N/A | Y | N/A |  |
| *ccna_00792* | Y | N | N/A |  |
| *ccna_00793* | Y | N | N/A |  |
| *ccna_01065* | N | N | N/A |  |
| *ccna_01066* | N | N | N/A |  |
| *ccna_01068* | N | N | N/A |  |
| *ccna_01104* | N | Y | N/A |  |
| *ccna_02035* | Y | N | *wcaL* | N |
| *ccna_03277* | Y | Y | *wcaL* | Y |
| *ccna_03455* | Y | Y | *wcaL* | N |
