# Supplementary material for: Caulobacter crescentus Adapts to Phosphate Starvation by Synthesizing Anionic Glycoglycerolipids and a Novel Glycosphingolipid
Source: mBio. 2019 Apr 2;10(2):e00107-19. doi: 10.1128/mBio.00107-19 (PMC6445935; doi:10.1128/mBio.00107-19)
Supplement: FIG S2 [file mBio.00107-19-sf002.pdf]

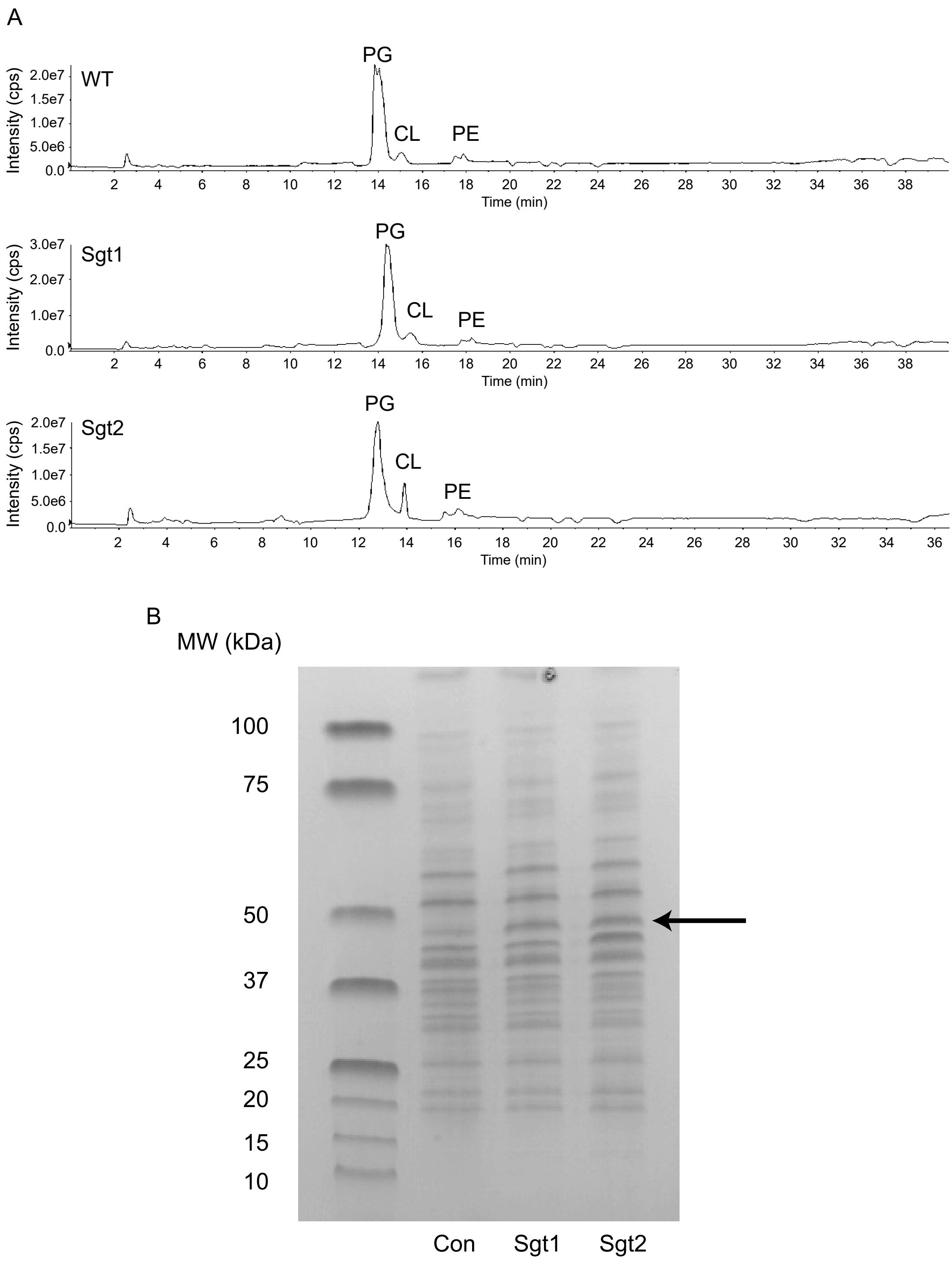

Figure S2. Sgt1 and Sgt2 specifically glycosylate ceramide lipids. (A) Total ion chromatograms of lipids from *E. coli* (strains MG1655, EK730, EK725) grown in LB media with 1 mM IPTG to induce Sgt1 and Sgt2 protein expression. The major phospholipid species phosphatidylglycerol (PG), cardiolipin (CL), and phosphatidylethanolamine (PE) were detected. No glycophospholipids were detected in Sgt overexpression strains. (B) SDS-PAGE gel of *E. coli* lysates confirms glycosyltransferase expression (arrow).
