# Supplementary material for: Caulobacter crescentus Adapts to Phosphate Starvation by Synthesizing Anionic Glycoglycerolipids and a Novel Glycosphingolipid
Source: mBio. 2019 Apr 2;10(2):e00107-19. doi: 10.1128/mBio.00107-19 (PMC6445935; doi:10.1128/mBio.00107-19)
Supplement: FIG S4 [file mBio.00107-19-sf004.pdf]

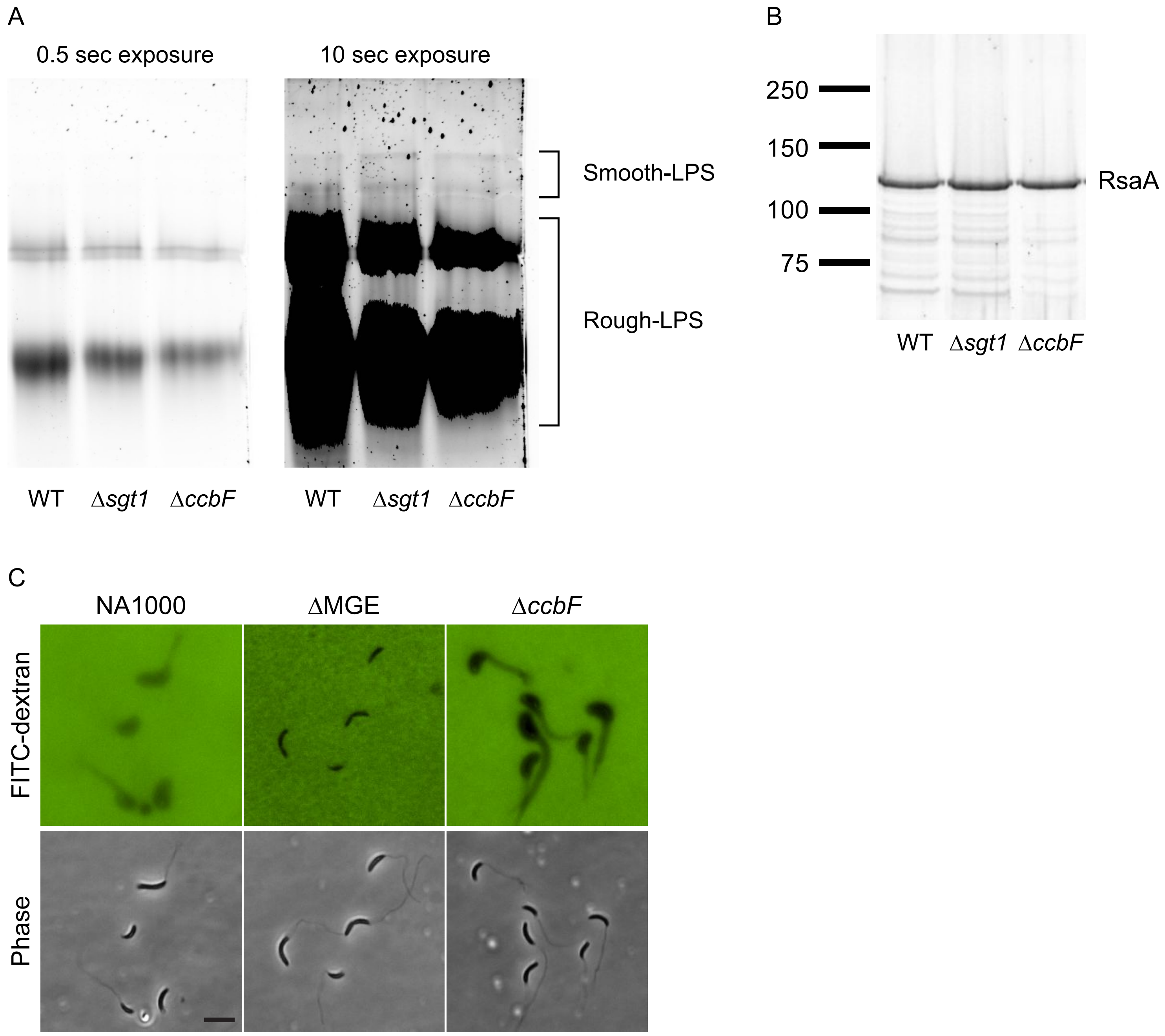

Figure S4. Ceramide deficiency does not alter LPS, S-layer, or EPS production. (A-B) LPS (A) and the S-layer protein RsaA (B) were purified from wild-type, Δ*sgt1, and* Δ*ccbF* cells grown in HIGG-1 μM phopsphate, and resolved by SDS-PAGE. (A) LPS was stained with Pro-Q Emerald 300. Since the rough-LPS is much more abundant, two different gel exposures are shown to demonstrate that ceramide deficiency has no significant effect on either smooth- or rough-LPS. (B) RsaA was stained with Krypton protein stain. (C) EPS production of *C. crescentus* strains was assessed by the size of the exclusion-zone of FITC-dextran. NA1000 ΔMGE is a non-EPS producing negative-control strain. Scale bar: 5 μm.
