# Supplementary material for: Caulobacter crescentus Adapts to Phosphate Starvation by Synthesizing Anionic Glycoglycerolipids and a Novel Glycosphingolipid
Source: mBio. 2019 Apr 2;10(2):e00107-19. doi: 10.1128/mBio.00107-19 (PMC6445935; doi:10.1128/mBio.00107-19)
Supplement: TABLE S1 [file mBio.00107-19-st001.docx]

**Table S1. Strains used in this study.**

| **Strain** | **Genotype** | **Construction** | **Source** |
| --- | --- | --- | --- |
| *C. crescentus* | | | |
| NA1000 | Synchronizable variant of wild-type *C. crescentus* strain CB15 |  | (1) |
| YB720 | NA1000 *phoBΩ12*; *phoB* deletion strain |  | (2) |
| EK717 | ΔMGE | Transformation of NA1000 with pEK729 | This study |
| EK720 | Δ*ccna_01220* | Transformation of NA1000 with pEK722 | This study |
| EK721 | Δ*ccna_01647* | Transformation of NA1000 with pEK723 | This study |
| EK724 | Δ*ccna_00792* | Transformation of NA1000 with pEK726 | This study |
| EK725 | Δ*ccna_00793* | Transformation of NA1000 with pEK727 | This study |
| GS64 | Δ*ccna_00792*;  PxylX:: *ccna_00792* | Transformation of EK724 with pGS62 | This study |
| GS65 | Δ*ccna_00793*;  PxylX:: *ccna_00793* | Transformation of EK725 with pGS63 | This study |
| GS66 | Δ*ccna_01220*;  PxylX:: *ccna_01220* | Transformation of EK720 with pGS61 | This study |
| GS78 | PvanA:: *ccna_00793* | Transformation of NA1000 with pGS77 | This study |
| GS80 | PxylX:: *ccna_00792* | Transformation of NA1000 with pGS79 | This study |
| GS81 | PxylX:: *ccna_00792;*  PvanA:: *ccna_00793* | Transduction of *vanA* locus into GS78 | This study |
| *E. coli* | | | |
| S17-1 | λ−pir cloning strain, Spec^R^ |  | (3) |
| XL1-Blue | Cloning strain, Tet^R^ |  | Agilent Technologies |
| MG1655 | Wild-type K-12 strain |  | (4) |
| EK730 | pTrc99d *ccna_00793* | Transformation of MG1655 with pEK731 | This study |
| EK735 | pTrc99d *ccna_00792* | Transformation of MG1655 with pEK734 | This study |
