# Supplementary material for: Caulobacter crescentus Adapts to Phosphate Starvation by Synthesizing Anionic Glycoglycerolipids and a Novel Glycosphingolipid
Source: mBio. 2019 Apr 2;10(2):e00107-19. doi: 10.1128/mBio.00107-19 (PMC6445935; doi:10.1128/mBio.00107-19)
Supplement: TABLE S2 [file mBio.00107-19-st002.docx]

Table S2. Plasmids used in this study.

| **Name** | **Description** | **Source** |
| --- | --- | --- |
| pNPTS138 | *sacB*-containing suicide vector used for double homologous recombination, Kan^R^ | Alley, M.R.K. (unpublished) |
| pEK722 | pNPTS138-based plasmid for deleting *ccna_01220*, Kan^R^ | This study |
| pEK723 | pNPTS138-based plasmid for deleting *ccna_01647*, Kan^R^ | This study |
| pEK726 | pNPTS138-based plasmid for deleting *ccna_00792*, Kan^R^ | This study |
| pEK727 | pNPTS138-based plasmid for deleting *ccna_00793*, Kan^R^ | This study |
| pEK729 | pNPTS138-based plasmid for deleting the mobile-genetic elecment (MGE), Kan^R^ | This study |
| pTrc99a | Ptac containing expression vector used for IPTG induced overexpression in *E. coli*, Amp^R^ | (5) |
| pTrc99d | pTrc99a-based vector with the backbone NdeI site removed and the NcoI site replaced with a new NdeI site, Amp^R^ | This study |
| pEK731 | pTrc99d-based plasmid for heterologous expression of CCNA_00793 in *E. coli*, Amp^R^ | This study |
| pEK734 | pTrc99d-based plasmid for heterologous expression of CCNA_00792 in *E. coli*, Amp^R^ | This study |
| pXCHYC-5 | Xylose-inducible expression, Tet^R^ | (6) |
| pXMCS-4 | Plasmid integrates at the *xylX* locus for xylose-inducible expression, Gent^R^ | (6) |
| pVCFPC-1 | Plasmid integrates at the *vanA* locus for vanillate-inducible expression, Spec^R^ | (6) |
| pGS61 | pXCHYC-5-based plasmid for *ccna_01220* expression, Tet^R^ | This study |
| pGS62 | pXCHYC-5-based plasmid for *ccna_00792* expression, Tet^R^ | This study |
| pGS63 | pXCHYC-5-based plasmid for *ccna_00793* expression, Tet^R^ | This study |
| pGS77 | pXMCS-4-based plasmid for *ccna_00793* expression, Gent^R^ | This study |
| pGS79 | pVCFPC-1-based plasmid for *ccna_00792* expression, Spec^R^ | This study |
