# Supplementary material for: Caulobacter crescentus Adapts to Phosphate Starvation by Synthesizing Anionic Glycoglycerolipids and a Novel Glycosphingolipid
Source: mBio. 2019 Apr 2;10(2):e00107-19. doi: 10.1128/mBio.00107-19 (PMC6445935; doi:10.1128/mBio.00107-19)
Supplement: TABLE S3 [file mBio.00107-19-st003.docx]

**Table S3. Primers used in this study.**

| **Name** | **Sequence** |
| --- | --- |
| *Cloning primers* | |
| EK779 | [phos]atggaattcgagctcggtaccc |
| EK780 | [phos]atgctgtttcctgtgtgaaattgttatccgc |
| EK785 | tactcatATGCGGATCGTGGACGTCGC |
| EK786 | tactaagcttTCATGGCCTCGCCTCTCCG |
| EK787 | tactcatATGCCTGCTCTAAGTCATGGGCTG |
| EK788 | tactaagcttCTAGGGCGTCGACAGCGC |
| EK820 | tacttctagaGCCGACAGATGGATCAGGT |
| EK821 | GGCGAAATGGAGCTTGCGGTCGATATAGG |
| EK822 | CGCAAGCTCCATTTCGCCGAACTCTTTGAG |
| EK823 | tactgaattcCGTCAGGACCAGGGTCAG |
| EK824 | tacttctagaGAGATAGCGCCAGAACCATT |
| EK825 | CGAGAATTTGGCTCAGCCCATGACTTAGA |
| EK826 | GGCTGAGCCAAATTCTCGACGAGGTGGTG |
| EK827 | tactgaattcTCGATCTCGGAGCTCTTGTT |
| EK897 | AAGCTTGGCGCCAGCCGG |
| EK898 | GAATTCGCTAGCTTCGGC |
| EK899 | gctggcgccaagcttTTTAGGCAAGGCTAGGGAG |
| EK900 | caggcgcgTAGCCCCATTAGTTAAGCC |
| EK901 | tggggctaCGCGCCTGAAACGGCGCT |
| EK902 | cgaagctagcgaattcATCGGTCCTGCCGACGCC |
| EK903 | gctggcgccaagcttCCCGGAAAGCCAGGAAC |
| EK904 | cacgaggcGCTCCGCATGTCATTTTCC |
| EK905 | tgcggagcGCCTCGTGAAATCGCTCTTTGTCGC |
| EK906 | cgaagctagcgaattcGGACAGGCCTCGCGCACG |
| EK957 | gctggcgccaagcttACGCCTCGAAGGCTGCGC |
| EK958 | tagcgctcGGTGGCCAACCGCCTCGATG |
| EK959 | tggccaccGAGCGCTAGACTCGATCC |
| EK960 | cgaagctagcgaattcGGCGACACCTTCCCCATG |
| EK975 | cgaggaaacgcatatgCCTGCTCTAAGTCATGGGCTG |
| EK976 | gggctgcaggaattcCTAGGGCGTCGACAGCGC |
| EK1019 | tggggagacgaccatATGCGGATCGTGGACGTC |
| EK1020 | cgggctgcagctagcTCATGGCCTCGCCTCTCC |
| EK1021 | tggggagacgaccatATGCCTGCTCTAAGTCATGGG |
| EK1022 | cgggctgcagctagcCTAGGGCGTCGACAGCGC |
| EK1023 | tggggagacgaccatATGGGGCTATTTGATAAGCACCTGGCCTATCGC |
| EK1024 | cgggctgcagctagcTCAGGCGCGGGCGCGCTT |
| *Primers for assessing gene deletions* | |
| EK S197 | CCAGCCCGTAGTAGTCCAGA |
| EK S198 | GCTGGTGACGAACAGGAGA |
| EK S199 | GACGGTGGTGTCGAAATGA |
| EK S200 | GATAGGCGTAGCGGCTGTAG |
| EK S216 | ACCCCGTCTGATAAGGCTTC |
| EK S217 | GCGAGACCGTGATCGACT |
| EK S218 | GGATCAGAGGCTCCACCAG |
| EK S219 | GCTAGGCCATAAGCAGATCG |
| EK S224 | CTGATCATCGCCGTGTTCTA |
| EK S225 | GCCACCTGAAGTACGAGACC |

| QPCR Primers | Forward | Reverse |
| --- | --- | --- |
| *rpoD* | CTCTATGCGATCAACAAGCG | ATAGGCCTTGAGGAACTCGC |
| *ccna_00792* | CCACGAGCTGTTCGTCATC | TGTAGTAGTTGGCGTCAAACG |
| *ccna_00793* | AGCCTTTGTCAGGACCAGAA | CACCACCTCGTCGAGAATTT |
| *ccna_01120* | ACAACGATCCCGAAGACTTG | ATCGCCGATCATCGAATAGA |
