# Supplementary material for: Caulobacter crescentus Adapts to Phosphate Starvation by Synthesizing Anionic Glycoglycerolipids and a Novel Glycosphingolipid
Source: mBio. 2019 Apr 2;10(2):e00107-19. doi: 10.1128/mBio.00107-19 (PMC6445935; doi:10.1128/mBio.00107-19)
Supplement: TEXT S1 [file mBio.00107-19-s0001.docx]

Supplementary methods

**Strain construction.** Creation of the various *C. crescentus* deletion strains was done by double-homologous recombination using both positive and negative selection. The suicide plasmid pNPTS138 has both a kanamycin resistance cassette as well as the *sacB* gene which is toxic in the presence of sucrose. Genomic fragments (500-1000 bp) upstream and downstream of the targeted gene were ligated in tandem in the pNPTS138 vector. The plasmid was transformed into *C. crescentus* and recombinants were selected on kanamycin plates. Individual colonies were grown overnight in PYE (without antibiotic) and streaked out onto PYE-3% sucrose plates to recover colonies that performed the second recombination. Colonies were screened for the gene deletion by PCR and streaked out onto plain PYE and PYE-kanamycin plates to confirm the loss of the plasmid backbone.

Strain EK720 (Δ*ccna_01220*) was cloned by Gibson assembly. pNPTS138 was linearized by PCR with primers EK897/898. The upstream (EK899/900) and downstream (EK901/902) homology fragments were PCR amplified from NA1000 genomic DNA. The assembled plasmid (pEK722) was electroporated into NA1000 followed by selection on PYE-kanamycin plates. An individual colony was grown overnight in PYE and streaked onto PYE-3% sucrose plates. Colonies were screened for the *ccna_01220* deletion with primers EK S216/S217 (wild-type 2.3kb; deletion 1.2 kb).

Strain EK721 (Δ*ccna_01647*) was cloned by Gibson assembly. pNPTS138 was linearized by PCR with primers EK897/898. The upstream (EK903/904) and downstream (EK905/906) homology fragments were PCR amplified from NA1000 genomic DNA. The assembled plasmid (pEK723) was electroporated into NA1000 followed by selection on PYE-kanamycin plates. An individual colony was grown overnight in PYE and streaked onto PYE-3% sucrose plates. Colonies were screened for the *ccna_01647* deletion with primers EK S218/S219 (wild-type 2.5kb; deletion 1.4 kb).

Strain EK724 (Δ*ccna_00792*) was cloned by PCR amplifying the upstream (EK820/821) and downstream (EK822/823) homology fragments NA1000 genomic DNA. The fragments were stitched together by overlap PCR. The final purified PCR product was cut with XbaI and EcoRI and ligated into the SpeI/EcoRI site of pNPTS138. The assembled plasmid (pEK726) was electroporated into NA1000 followed by selection on PYE-kanamycin plates. An individual colony was grown overnight in PYE and streaked onto PYE-3% sucrose plates. Colonies were screened for the *ccna_00792* deletion with primers EK S197/S198 (wild-type 2.4kb; deletion 1.2 kb).

Strain EK725 (Δ*ccna_00793*) was cloned by PCR amplifying the upstream (EK824/825) and downstream (EK826/827) homology fragments NA1000 genomic DNA. The fragments were stitched together by overlap PCR. The final purified PCR product was cut with XbaI and EcoRI and ligated into the SpeI/EcoRI site of pNPTS138. The assembled plasmid (pEK727) was electroporated into NA1000 followed by selection on PYE-kanamycin plates. An individual colony was grown overnight in PYE and streaked onto PYE-3% sucrose plates. Colonies were screened for the *ccna_00793* deletion with primers EK S199/S200 (wild-type 2.4kb; deletion 1 kb).

Strain EK717 (Mobile-genetic element deletion; ΔMGE) was cloned by PCR amplifying the upstream (EK957/958) and downstream (EK959/960) homology fragments NA1000 genomic DNA. The plasmid backbone (pNPTS138) was linearized with primers EK897/898 and the three components were assembled by Gibson assembly. The resulting plasmid (pEK729) was electroporated into NA1000 followed by selection on PYE-kanamycin plates. An individual colony was grown overnight in PYE and streaked onto PYE-3% sucrose plates. Colonies were screened for the MGE deletion with primers EK S224/S225 (wild-type no product; deletion 2.3 kb).

Strain EK730 (*E. coli* CCNA_00793 overexpression) was produced by electroporating plasmid pEK731 into MG1655. pEK731 was constructed by first removing the NdeI site in the pTrc99a backbone by digesting pTrc99a with NdeI, blunting the overhangs with the DNA polymerase Klenow fragment, and religating the blunt ends. Next, the NcoI restriction site in pTrc99a was replaced with an NdeI site by inverse PCR using primers EK779/780 resulting in plasmid pTrc99d. *ccna_00793* was cloned using primers EK787/788 and ligating into the NdeI/HindIII site of pTrc99d.

Strain EK735 (*E. coli* CCNA_00792 overexpression) was produced by electroporating plasmid pEK734 into MG1655. pEK734 was constructed by cloning *ccna_00792* using primers EK785/786 and ligating into the NdeI/HindIII site of pTrc99d.
 Strain GS64 (Δ*ccna_00792* complementation strain) was produced by electroporating plasmid pGS62 into EK724. Plasmid pGS62 was constructed by PCR-amplifying *ccna_00792* using primers EK1019/1020 and ligating into the NdeI/NheI site of pXCHYC-5.

Strain GS65 (Δ*ccna_00793* complementation strain) was produced by electroporating plasmid pGS63 into EK725. Plasmid pGS62 was constructed by PCR-amplifying *ccna_00793* using primers EK1021/1022 and ligating into the NdeI/NheI site of pXCHYC-5.
 Strain GS66 (Δ*ccna_01220* complementation strain) was produced by electroporating plasmid pGS61 into EK720. Plasmid pGS62 was constructed by PCR-amplifying *ccna_01220* using primers EK1023/1024 and ligating into the NdeI/NheI site of pXCHYC-5.

Strain GS78 (*ccna_00793* overexpression strain) was produced by electroporating plasmid GS77 into NA1000. Plasmid pGS77 was constructed by PCR-amplifying *ccna_00793* using primers EK975/976 and ligating into the NdeI/EcoRI site of pXMCS-4.

Strain GS80 (*ccna_00792* overexpression strain) was produced by electroporating plasmid GS79 into NA1000. Plasmid pGS79 was constructed by PCR-amplifying *ccna_00792* using primers EK1019/1020 and ligating into the NdeI/NheI site of pVCFPC-1.

Strain GS81 (*ccna_00792*/*ccna_00793* overexpression strain) was constructed by phage transduction (ΦCr30) using GS80 as the donor strain and GS79 as the recipient strain.

**Supplemental References**

1. Evinger M, Agabian N. 1977. Envelope-associated nucleoid from *Caulobacter crescentus* stalked and swarmer cells. J Bacteriol 132:294-301.
2. Gonin M, Quardokus EM, O'Donnol D, Maddock J, Brun YV. 2000. Regulation of stalk elongation by phosphate in *Caulobacter crescentus*. J Bacteriol 182:337-347.
3. Simon R, Priefer U, Puhler A. 1983. A broad host range mobilization system for in vivo genetic engineering: transposon mutagenesis in Gram negative bacteria. Bio/Technology 1:784.
4. Blattner FR, Plunkett G, 3rd, Bloch CA, Perna NT, Burland V, Riley M, Collado-Vides J, Glasner JD, Rode CK, Mayhew GF, Gregor J, Davis NW, Kirkpatrick HA, Goeden MA, Rose DJ, Mau B, Shao Y. 1997. The complete genome sequence of *Escherichia coli* K-12. Science 277:1453-62.
5. Amann E, Ochs B, Abel KJ. 1988. Tightly regulated tac promoter vectors useful for the expression of unfused and fused proteins in *Escherichia coli*. Gene 69:301-15.
6. Thanbichler M, Iniesta AA, Shapiro L. 2007. A comprehensive set of plasmids for vanillate- and xylose-inducible gene expression in *Caulobacter crescentus*. Nucleic Acids Res 35:e137.
7. Lubin EA, Henry JT, Fiebig A, Crosson S, Laub MT. 2016. Identification of the PhoB regulon and role of PhoU in the phosphate starvation response of *Caulobacter crescentus*. J Bacteriol 198:187-200.
8. Christen B, Abeliuk E, Collier JM, Kalogeraki VS, Passarelli B, Coller JA, Fero MJ, McAdams HH, Shapiro L. 2011. The essential genome of a bacterium. Mol Syst Biol 7:1-7.
9. Altschul SF, Gish W, Miller W, Myers EW, Lipman DJ. 1990. Basic local alignment search tool. J Mol Biol 215:403-10.
